# A review of attitude research that is specific, accurate, and comprehensive within its stated scope: responses to Aarons

**DOI:** 10.1186/s13012-022-01200-z

**Published:** 2022-05-10

**Authors:** Jessica Fishman, Catherine Yang, David S. Mandell

**Affiliations:** 1 Perelman School of Medicine, University of Pennsylvania, 3535 Market Street, Philadelphia, PA 19104 USA; 2 Message Effects Lab at Annenberg School, University of Pennsylvania, 3620 Walnut Street, Philadelphia, PA 19104 USA

Dear Editors-in-Chief (*Implementation Science*):

Thank you for the opportunity to respond to Dr. Aarons’ letter regarding our article *Attitude theory and measurement in implementation science: a secondary review of empirical studies and opportunities for advancement* [1]. Dr. Aarons shares three main concerns with our review: (1) that there was a missing attribution to him, as the creator of the EBPAS; (2) whether the EBPAS measures attitudes; and (3) if our review should have included additional studies using the EBPAS. Below, we address each.

First, Dr. Aarons states that we should have made an attribution to him when referencing the developers of the EBPAS. We did cite Aarons and colleagues in the version of the manuscript that was accepted for publication; it appears the journal mistakenly changed the reference. We hope that this can be rectified and thank Dr. Aarons for bringing it to our attention.

Secondly, we respectfully disagree with Dr. Aarons about whether the EBPAS measures attitudes. As defined in the social psychology literature from which the term emanates, an attitude towards a behavior, such as using an evidence-based practice, refers to how strongly one believes that performing that behavior would have favorable or unfavorable consequences [2–4]. In implementation science, one’s attitudes towards a particular evidence-based practice would represent the perceived advantages and disadvantages of doing so [2–4]. There are many published methodological accounts of how to adapt validated measurement approaches, which differ fundamentally from EBPAS items and response options.

In the 15-item version of the EBPAS [5], almost all items deviate conceptually from an attitude. As an example, several items ask respondents to report “how likely” they are to use EBP under different circumstances. In psychology, such items would be considered conceptually similar to behavioral intention, not attitudes [6, 7]. The more recent 36-item version of the EBPAS [8] also includes items that are conceptually closer to other psychological constructs. For example, the following item is conceptually related to self-efficacy: “I don’t know how to fit evidence-based practice into my administrative work.” We do not meant to diminish the importance of measuring constructs other than attitudes, but it is useful to distinguish between distinct psychological constructs, which have different roles in causal models predicting and changing behavior.

We also disagree about the importance of measuring attitudes towards specific behaviors rather than general categories of behaviors. EBPAS items refer to general categories of behavior, such as trying “new practices,” “evidence-based practices,” or “evidence-based treatment” [5, 8]. Yet, over several decades, a large attitude literature in psychology has empirically demonstrated the advantages of measuring attitudes towards a specific behavior, rather than general categories of behavior [2–4].

Consistent with the results from psychology, the implementation science literature has started to document how practitioners’ attitudes can vary greatly among evidence-based practices [9–12]. For example, we have found that therapists’ attitudes vary towards different components of cognitive-behavioral therapy [10]. Given this variability, a measure of attitudes towards “evidence-based practice” or even “cognitive behavioral therapy” would sacrifice psychometric performance, including predictive validity [9–12]. Depending on the specific evidence-based practice, other psychological variables also can vary [9–12].

A related concern is that practitioners often lack familiarity with the phrase “evidence-based practice,” as Dr. Aarons and colleagues have acknowledged [5, 8]. The EBPAS directions state that “evidence-based practice” refers to any intervention that is supported by “empirical research,” but as Dr. Aarons and colleagues acknowledge, practitioners may still be confused, due to a lack of knowledge [5, 8]. For example, Aarons wrote, “Familiarity with the term ‘evidence-based practice’ among program managers was low” [5]. He added that respondents had “only a low level of familiarity with even the terminology of EBP,” including the descriptor “empirically supported treatment” [5]. Additionally, practitioners may not know which practices have been designated as “evidence-based,” “research-based,” or “empirically supported.” Depending on one’s knowledge, responses to the EBPAS may differ, which is problematic if the goal is to measure attitudes.

Finally, Dr. Aarons points out that we did not include many studies that use the EBPAS. The EBPAS was featured only briefly in our review because our review was not focused on the EBPAS. Dr. Aarons suggests that our study selection was biased. We are surprised by this concern because we explicitly stated the inclusion and exclusion criteria, which relied on a rigorous, systematic review (authored by Aarons and colleagues [13]), and we adhered to the criteria.

We agree with Dr. Aarons that future reviews could change the inclusion criteria and generate a different sample of studies. Indeed, in our review [1], we called for this additional research, and we welcome the replication with a different sample. Dr. Aarons correctly notes that there are thousands of articles that could be reviewed if different inclusion criteria were used. He suggests that the studies we reviewed are not representative of all implementation studies that are concerned with attitudes. Since we lack reviews of how these other implementation studies define or measure attitudes, whether our results are representative is an open question.

Implementation science has been described as “somewhat elusive” because it has not yet developed distinct construct definitions [14]. Our review documents conceptual ambiguity and suggests that a definition of attitudes (from psychology) could be useful for implementation research [1]. Our review also provides specific examples of how implementation scientists measure attitudes in ways that differ from each other and from validated approaches used in social psychology. As implementation science strives to develop standardized measurement approaches, some of the rigorously developed methods from social psychology could offer valuable scientific opportunities.

## Data Availability

The data generated or analyzed for the review are included in Fishman, J., Yang, C. & Mandell, D. Attitude theory and measurement in implementation science: a secondary review of empirical studies and opportunities for advancement. *Implementation Sci* 16, 87 (2021).
